# Reduction of cell viability and alteration of genomic DNA methylation by phthalates in a normal human fibroblast (NHF) cell line *invitro*

**DOI:** 10.1016/j.bbrep.2026.102755

**Published:** 2026-08-24

**Authors:** Warqaa Y. Salih, Fikrat M. Hassan, Majeed A. Sabbah

**Affiliations:** a Institute of Genetic Engineering and Biotechnology for Post Graduate Studies, University of Baghdad, Iraq; b Department of Biology, College of Science for Women, University of Baghdad, Baghdad, Iraq; c Forensic DNA Research and Training Center, Al-Nahrain University, Jadriya, Baghdad, Iraq

**Keywords:** Phthalates, NHF, MTT assay, Cell viability, ELISA, Genomic methylation

## Abstract

Phthalates are ubiquitous environmental contaminants widely used as plasticizers in poly vinyl chloride products, and growing evidence implicates them in metabolic activity reduction and epigenetic dysregulation. However, their direct effects on non-transformed human cells remain incompletely characterized. The present *in vitro* study evaluated the reduction in cell viability and DNA methylation–modulating effects of three widely distributed phthalates, di(2-ethylhexyl) phthalate (DEHP), di-n-butyl phthalate (DnBP), and di-isononyl phthalate (DINP), on normal human fibroblast (NHF) cells. Cells were exposed to serial concentrations (31.25–1000 μg/mL) for 72 h, and the viability was assessed by MTT assay, followed by IC_50_ determination via nonlinear regression. Global DNA methylation was subsequently quantified at the IC_50_ concentration by ELISA-based measurement of 5-methylcytosine (5-mC). All three phthalates produced significant concentration-dependent reductions in cell viability. Constraining the regression plateaus to 100% and 0% viability yielded estimated IC_50_ values of 19.2 μg/mL for DEHP, 43.4 μg/mL for DnBP, and 13.1 μg/mL for DINP; as viability fell below 50% at the lowest concentration tested, these values represent extrapolation below the sampled range, their confidence intervals overlap, and no potency ranking is asserted. At fixed high-effect concentrations, DEHP and DINP significantly elevated global 5-mC levels to 189.18% and 159.56% of control, respectively, whereas DnBP induced only a non-significant increase (120.06%). These findings indicate that phthalates compromise both the viability and epigenetic integrity of NHF cells in a congener-specific manner, underscoring their potential contribution to methylation-associated human pathologies.

## Highlights

• All phthalate compounds exhibited cell viability loss, with different capacities to induce metabolic activity reduction.
• Exposure to phthalate compounds resulted in specific effects in DNA methylation levels.
• Di(2-ethylhexyl) phthalate and di-isononyl phthalate significantly increased the DNA methylation levels.

## Graphical abstract

**Figure:**
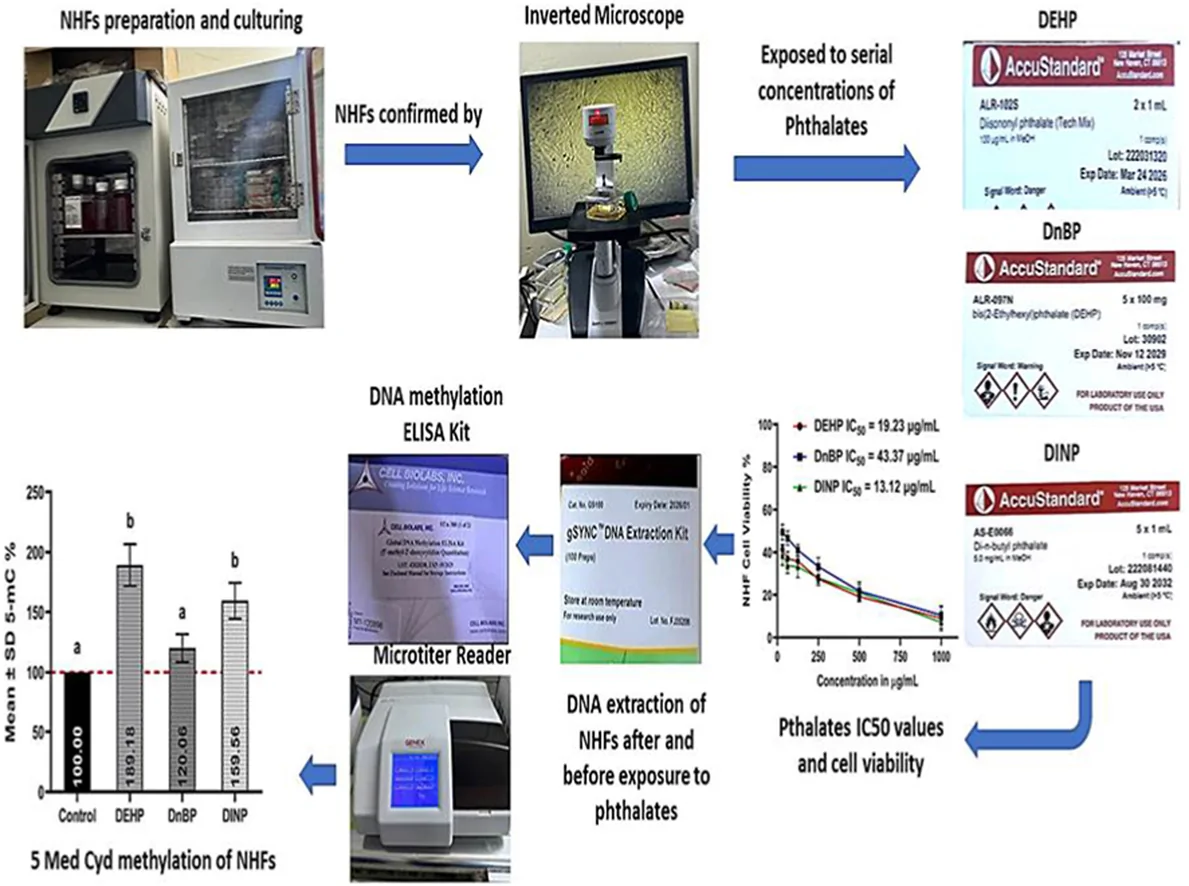

## 1 Introduction

Phthalates constitute a class of synthetic chemicals widely employed in industrial applications, most notably as plasticizers in polyvinyl chloride (PVC). Owing to the immense scale of global plastic production, these compounds have become ubiquitous environmental contaminants. Studies have increasingly documented the harmful effects of phthalates on human physiology, particularly on the endocrine and reproductive systems [1].

Plasticizers are low-molecular-weight additives incorporated into polymers such as PVC to make the plastic softer and more flexible, thereby enhancing its plasticity, decreasing its viscosity, and/or reducing the friction associated with handling [2,3]. Several classes of chemicals were used as plasticizers in the early 20th century. Currently, more than 100 different plasticizers are produced worldwide, half of which are in common use [4].

Phthalates are conventionally classified on the basis of their chemical structure, with dibasic acid esters and glycol derivatives representing the predominant categories [5]. Because plasticizers are physically dispersed within, rather than covalently bonded to, the polymer matrix, they readily migrate from finished products into the surrounding environment. Consequently, phthalates have been detected across diverse environmental compartments, including surface waters, soils, sediments, indoor dust, and wastewater effluents [6]. Human exposure is further compounded by their migration into foodstuffs, particularly lipid-rich matrices such as dairy products, meat, and fish, rendering dietary intake a routine and unavoidable route of exposure [7,8]. Low-molecular-weight phthalates (LMWPs) can enter the human body via three principal routes: inhalation, ingestion, and dermal absorption. Children are particularly vulnerable to the adverse effects of these compounds, as early-life exposure may impair developmental and reproductive processes and precipitate endocrine disruption [9].

Phthalic acid esters (PAEs) are extensively employed in everyday products owing to their favorable physicochemical properties in food-contact applications [10]. Their adverse effects on human health have been widely investigated, with exposure implicated in neurological disorders and a range of systemic complications [1,11]. Exposure may also occur in utero, as certain phthalates are capable of crossing the placental barrier and reaching the developing embryo [12].

In humans, normal dermal fibroblasts synthesize extracellular matrix components such as collagen and elastin, which are essential for connective tissue development, tissue regeneration, and wound healing. Given their pivotal role in maintaining tissue integrity and their responsiveness to cellular stress, normal human fibroblasts (NHFs) are commonly employed to assess phthalate-induced cell viability loss *in vitro* [13,14]. Several phthalate esters, di(2-ethylhexyl) (DEHP), butyl benzyl, and dimethyl phthalates, have been detected throughout the biosphere, raising concerns regarding both ecological integrity and public health [9]. Direct exposure to DEHP and its active metabolite mono-2-ethylhexyl phthalate (MEHP) has been shown to alter the expression of peroxisome proliferator-activated receptors (*PPARα* and *PPARγ*), key regulators of fatty acid metabolism, adipocyte proliferation, and adipose tissue development, thereby disrupting metabolic homeostasis and promoting obesity [15,16]. Consistent with these findings, environmental phthalate exposure has been epidemiologically linked to increased body weight, hormonal dysregulation, and elevated cardiovascular disease risk [17,18].

Epigenetics encompasses heritable modifications to DNA structure that alter transcriptional activity without changing DNA sequence, with DNA methylation representing the most prominent example in humans [19]. Aberrant methylation can dysregulate gene expression, compromise genomic stability and drive tumorigenesis through the activation or silencing of key proteins [20].

DNA methylation is a central epigenetic mechanism governing gene expression, genomic stability, and developmental programming in which DNA methyltransferases catalyze the addition of methyl groups to cytosine residues at CpG sites [21]. Mounting evidence indicated that phthalate exposure perturbed global and gene-specific methylation patterns, driving aberrant transcriptional silencing and compromising genomic integrity [11,22]. Such disruptions have been mechanistically linked to adverse health outcomes, including impaired neurodevelopment and neurological disease [23], as well as the initiation and progression of malignancies, underscoring phthalates as environmental contributors to the dysregulation of the human methylome [24].

Despite the evidence of phthalates implication in epigenetics dysregulation, the direct effect on cell viability and methylation-modulating effects of individual phthalates on non-transformed human cells remain incompletely characterized. The present *in vitro* study was therefore designed to evaluate the cell viability of three widely distributed phthalates, di(2-ethylhexyl) phthalate (DEHP), di-n-butyl phthalate (DnBP), and di-isononyl phthalate (DINP), against normal human fibroblast (NHF) cells and to determine their capacity to induce global methylation changes. The concentrations tested (31.25–1000 μg/mL) exceed typical human exposures and were selected for an acute, high-dose comparative screen of congener responses, not to model environmental exposure.

## 2 Materials and methods

### 2.1 Cell line and culture conditions

NHF normal human adipose tissue–derived mesenchymal stem cells was established in Iraqi Center for Cancer Research and Medical Genetics - Mustansiriyah University from human adipose tissue obtained from a healthy donor and kindly provided in 25 cm^2^ tissue culture flasks and was certified by the supplying center as free from microbial contamination prior to delivery. Derivation and use of the line were approved by the Institutional Ethics Committee of the ICCRMG (approval no. ICCRMG-79, dated 1 November 2024), and the tissue was obtained with the donor's informed consent in accordance with the Declaration of Helsinki. The line is not commercially available and has no assigned catalogue number. Cultures were routinely monitored by phase-contrast microscopy throughout the study and showed no evidence of bacterial or fungal contamination. Cells were used between passages 6 - 10 and were maintained in the exponential growth phase throughout using minimum essential medium (MEM) (US Biological, USA) supplemented with 10% (*v*/*v*) fetal bovine serum (FBS) (Capricorn Scientific, Germany), 100 IU/mL penicillin, 100 μg/mL streptomycin (Capricorn Scientific, Germany), 10 ng/mL human recombinant growth factor, and 4 μL heparin (0.2%). The cultures were incubated at 37°C in a humidified atmosphere containing 5% CO_2_. Cells in the exponential growth phase were used for subsequent experiments [25].

### 2.2 Preparation of phthalates

Phthalate esters (DEHP, DnBP, or DINP) were purchased from Accustandard (USA) and prepared individually by dissolving each compound in 100% dimethyl sulfoxide (DMSO) to yield a stock solution of 500 mg/mL. Preparations were sterilized by filtration using 0.22 μm Millipore filter and stored at −20°C. For dose-response assessment, twofold serial dilutions (1000, 500, 250, 125, 62.5, and 31.25 μg/mL) were prepared from working stock using sterilized MEM as diluent. The final DMSO concentration in the treatment wells ranged from 0.20% (v/v) at 1000 μg/mL to 0.006% at 31.25 μg/mL.

### 2.3 Cell viability

To investigate the cellular viability upon DEHP, DnBP, or DINP treatments, 3-(4,5-di methyl thiazol-2-yl)-2,5-diphenyltetrazolium bromide (MTT) assay was performed according to previously reported protocol [26]. Briefly, NHF cells at density 1 × 10^4^ were seeded in 96-well plates (NEST Biotech, China) and incubated at 37°C for 24 h. After incubation, medium was replaced with fresh MEM (100 μL/well) containing serial concentrations of DEHP, DnBP, or DINP. Cells were incubated for additional 72 h. Cells without treatment were considered negative control. Following incubation period, 10 μL of MTT solution (2 mg/mL) was added to each well and incubated at 37°C for 3 h. Then, 100 μL of DMSO was added to each well to solubilize formazan crystals, and the mixture was incubated again for an additional 15 min. The absorbance was measured at 570 nm using microplate reader (Gennex Lab, USA). The experiment was performed in triplicate and half maximum inhibitory concentration (IC_50_) for each phthalate was determined. Untreated cells maintained in medium alone served as the negative control. The cell viability rate (%) was calculated according to the following equation:

Viability Rate (%) = (OD Control – OD sample)/OD Control × 100

### 2.4 Global DNA methylation

#### 2.4.1 Genomic DNA extraction

Genomic DNA was extracted from NHF cells treated with DEHP, DnBP, or DINP at IC_50_ concentration for 72 h using the protocol of gSYNC™ DNA extraction kit (Geneaid Biotech, China).

#### 2.4.2 DNA methylation assay

Quantitative analysis of 5-methylcytosine (5-mC) level in the extracted genomic DNA was carried out via a Global DNA Methylation Kit (Abcam, Canada) in accordance with the manufacturer's instructions. In brief, genomic DNA samples were normalized to 5 ng/μL and 20 μL of each sample was mixed with 80 μL of binding solution in individual wells, followed by incubation at 37°C for 90 min. After the subsequent washing steps, an anti-5-mC antibody was applied, and the enzymatic reaction was terminated by addition of a stop solution. The resulting colorimetric signal was quantified at 450 nm using a microplate reader. The absolute quantity of methylated DNA was determined from a standard curve constructed by plotting the optical density (OD) values against the corresponding concentrations of the standard, from which the slope (OD/ng) was derived by linear regression according to the following equation:

$$-mC\;\left(ng\right)=\frac{\text{Sample}\;\text{OD}-ME3\;OD}{\text{Slope}\times 2}$$

## 3 Statistical analysis

Statistical analyses were performed using GraphPad Prism software version 9.0 (GraphPad Software Inc., LaJolla, CA). One Way ANOVA followed by post hoc Tukey's test was used to determine whether group variance between different concentrations within a treatment was significant or not. Data were expressed as mean ± SD and statistical differences were defined as *p* < 0.05.

## 4 Results

### 4.1 Effects of DEHP, DnBP and DINP against NHF viability

**Fig. 1:**
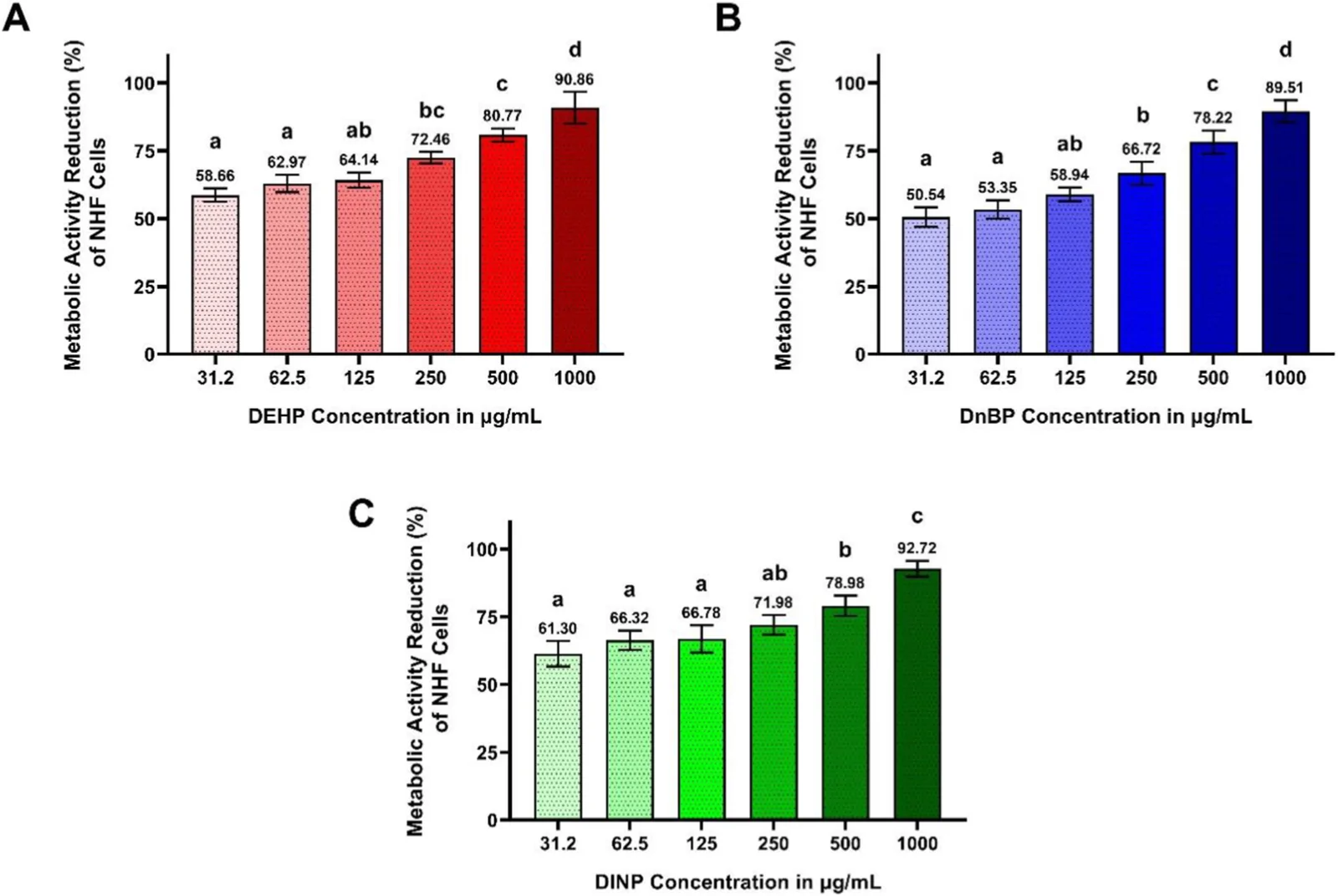
Concentration-dependent metabolic activity reduction effects of DEHP (A), DnBP (B), and DINP (C) on normal human fibroblasts (NHFs) following 72 h exposure, assessed by MTT assay. Values represent the mean ± SD of three independent experiments. Different letters (a, b, c, d) considered significantly different at *p* < 0.05. *n* = 3.

Exposure of NHF cells to DEHP, DnBP and DINP elicited a noticeable, concentration-dependent decrease in cell viability across the tested range of 31.2–1000 μg/mL (Fig. 1A–C). For all three compounds, viability decreased progressively with increasing concentration, with the highest dose (1000 μg/mL) producing the greatest cellular damage in each case.

DEHP (Fig. 1A) induced a metabolic activity radaction of 58.66 ± 2.5% at 31.2 μg/mL, which increased progressively at 62.5, 125, 250, and 500 μg/mL, reaching a maximum of 90.86 ± 5.82% at 1000 μg/mL. The values recorded at 31.2 and 62.5 μg/mL did not differ significantly, whereas the 500 and 1000 μg/mL treatments produced significantly (*p* < 0.0001) greater loss of cellular viability than all lower concentrations. DnBP (Fig. 1B) exhibited a comparable dose-response pattern, with viability decreasing from 50.54 ± 3.62% at 31.2 μg/mL to 53.35 ± 2.28%, 58.94 ± 2.55%, 66.72 ± 4.29%, and 78.22 ± 4.30% at 62.5, 125, 250, and 500 μg/mL, respectively, and peaking at 89.51 ± 4.09% at 1000 μg/mL. The concentrations 500 and 1000 μg/mL emerged as significantly (*p* < 0.0001) distinct among other concentrations. Finally, DINP (Fig. 1C) similarly produced dose-dependent loss of cellular viability against NHF cells, with values of 61.30 ± 4.72%, to 78.98 ± 3.81% cell inhibition at 31.2 to 500 μg/mL, respectively, culminating in 92.72% at 1000 μg/mL, the highest metabolic activity reduction capabilities recorded across all three compounds. The three lowest concentrations (31.2–125 μg/mL) showed no significant differences with respect to NHF loss of cell viability, whereas the 500 and 1000 μg/mL treatments were significantly distinct (*p* < 0.0001).

**Fig. 2:**
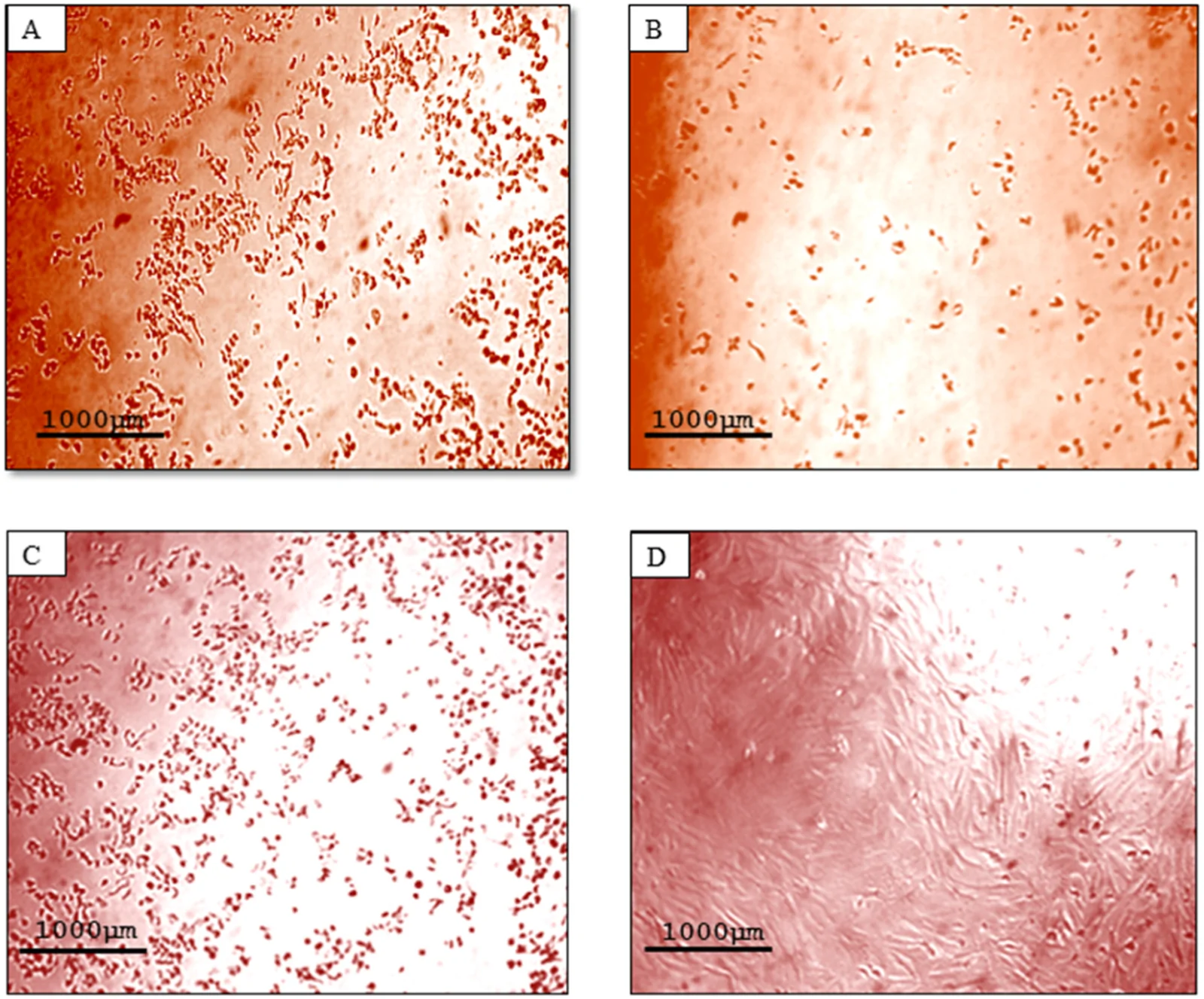
Microscopic images of NHF cells cultured in MEM supplemented with FBS (10%) and exposed to phthalates for 72 h at 1000 μg/mL. A: Cells treated with DEHP; B: cells treated with DnBP; C: cells treated with DINP; D: untreated cells (control). All the images were captured at 10X magnification, and the scale bar represents 1000 μm.

Morphologically, exposure to phthalates at 1000 μg/mL was accompanied by visible alterations in monolayer appearance Fig. (2 A-D) These included cell shrinkage, rounding, membrane blebbing, and detachment from the substratum. These observations are qualitative and corroborative; the mode of cell death was not determined, and no apoptosis-specific marker was assessed.

**Fig. 3:**
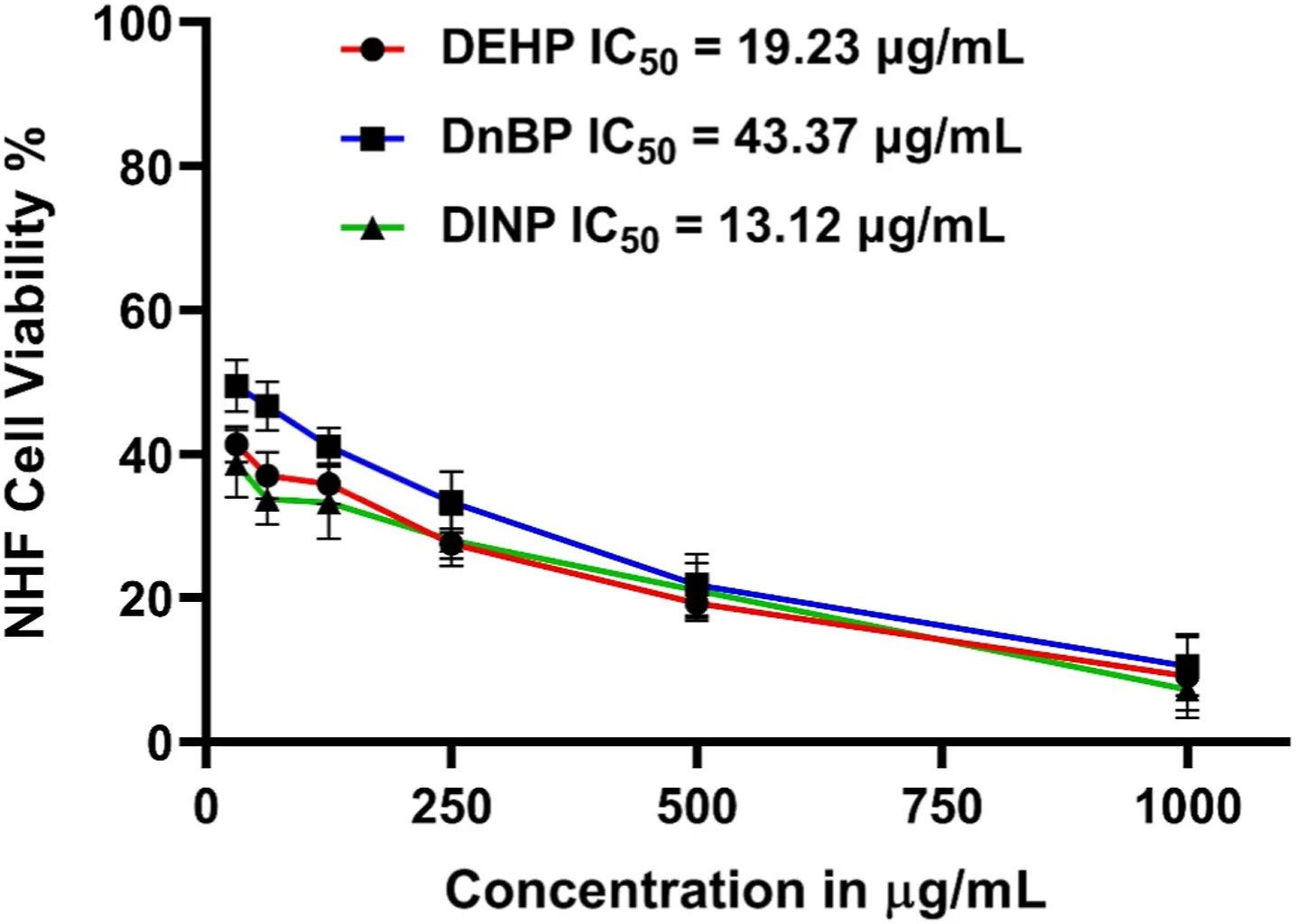
Dose–response curves and half-maximal inhibitory concentrations (IC_50_) of DEHP, DnBP, and DINP in normal human fibroblasts (NHFs) following 72-h exposure. Viability was assessed by MTT assay and expressed as the percentage of untreated control cells. IC_50_ values were calculated by nonlinear regression from three independent experiments. Data are presented as mean ± SD.

Four-parameter nonlinear regression of the dose–response data, with the upper and lower plateaus constrained to 100% and 0% viability, yielded model-estimated IC_50_ values of 13.12 μg/mL (95% CI: 5.39–31.95) for DINP, 19.23 μg/mL (95% CI: 10.73–34.47) for DEHP, and 43.37 μg/mL (95% CI: 29.90–62.92) for DnBP. Because viability fell below 50% at the lowest concentration tested, these estimates represent extrapolation below the sampled concentration range, and their confidence intervals overlap. Consequently, the IC_50_ estimates were not used to establish a comparative potency ranking. The dose-response data showed concentration-dependent reductions in MTT-derived metabolic activity for all three phthalates across the tested concentration range(Fig. 3).

### 4.2 Quantification of genomic methylation

**Fig. 4:**
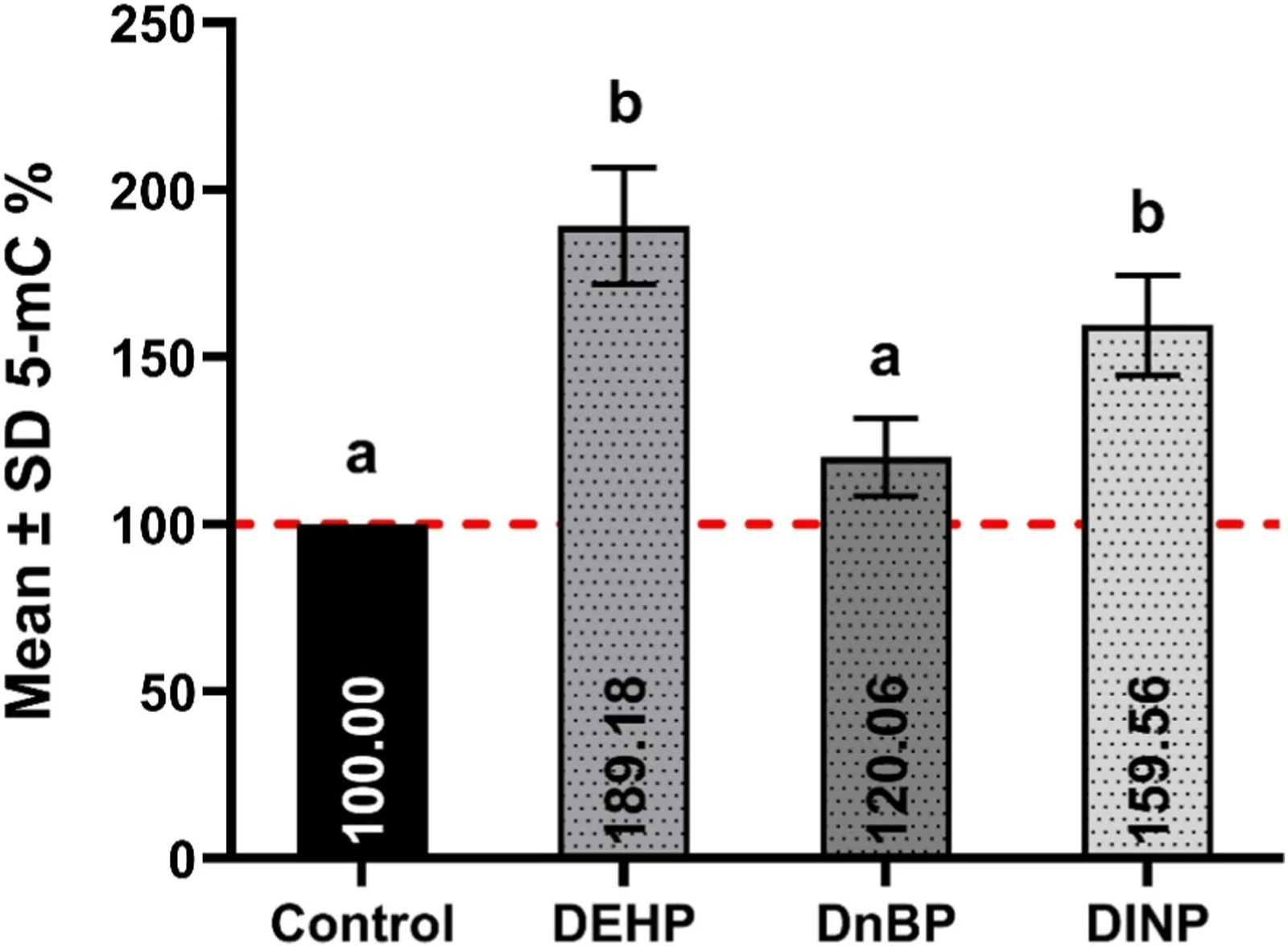
Global DNA methylation (5-methylcytosine, 5-mC) levels in normal human fibroblasts (NHFs) following treatment with DEHP, DnBP, or DINP at IC_50_ concentration. Values are expressed as a percentage of the untreated control (dashed red line, 100%) and presented as mean ± SD of three independent experiments. Bars labeled with different letters differ significantly (*p* < 0.05).

Global DNA methylation was assessed via ELISA, and the corresponding percentage of 5-mC was measured in the genomic DNA of untreated NHF cells as well as NHF cells treated with DEHP, DnBP and DINP at IC_50_ concentration for 72 h (Fig. 4). DEHP elicited the most pronounced effect, elevating 5-mC levels to 189.18 ± 17.36% of control, an approximately 1.9-fold increase, followed by DINP, which produced a 1.6-fold elevation (159.56 ± 14.93%). Both increases were statistically significant relative to untreated control (*p* = 0.0001 and 0.0021, respectively). In contrast, DnBP treatment resulted in a comparatively modest rise in methylation (120.06%), which did not differ significantly from the control group, suggesting that DnBP exerts a substantially weaker epigenetic effect on NHF cells under the conditions tested.

## 5 Discussion

Phthalates such as DEHP, DnBP, and DINP are widely used plasticizers that can leach from plastics into the environment and human body, leading to various health risks. Exposure to these chemicals has been linked to oxidative stress, endocrine disruption, reproductive toxicity, neurodevelopmental issues, and cancer risk [27]. In the liver, DEHP and DnBP cause mitochondrial damage, fat accumulation, and peroxisome proliferation, which can lead to hepatocyte enlargement and altered detoxification enzyme expression, contributing to liver damage [28]. Phthalate exposure is also associated with increased hypertension risk mediated by oxidative stress markers, suggesting cardiovascular implications [29]. Mono(2-ethylhexyl) phthalate (MEHP) induces neuronal death, oxidative damage, and synaptic dysfunction, potentially driving neurodevelopmental disorders [30], while dibutyl phthalate (DBP) elicits dose- and time-dependent metabolic activity reduction in human lymphocytes [31]. Two alternative plasticizers DINP and 1,2-cyclohexane dicarboxylic acid (DINCH) have been considered by manufacturers as a replacement for DEHP in many PVC products, such as medical devices and food packaging, but are limited in toys with concentrations inferior to 0.1% by weight of the plasticized material, due to their physicochemical properties suggesting a lower migration and more favorable toxicological profile than DEHP [32].

The present study showed a concentration-dependent reduction in MTT-derived metabolic activity in NHFs following exposure to DEHP, DnBP, and DINP. Non-linear regression yielded model-estimated IC_50_ values of 19.23, 43.37, and 13.12 μg/mL, respectively. The non-linear regression yielded different model-estimated IC_50_ values for the three congeners. Because the estimated values of DINP and DEHP fell below the lowest experimentally tested concentration and the confidence intervals overlapped, these estimates are reported as model-derived parameters and were not used for comparative interpretation. The observed concentration-dependent responses are broadly consistent with earlier comparative work examining high-molecular-weight branched phthalates in fibroblast models [33]. The progressive, dose-dependent decline in viability observed across the 31.2–1000 μg/mL range is consonant with the well-documented mechanistic sequence of phthalate-induced metabolic activity reduction: reactive oxygen species (ROS) accumulation, glutathione depletion, loss of mitochondrial membrane potential, cytochrome *c* release, and activation of the intrinsic apoptotic pathway, a cascade demonstrated for MEHP in human endothelial cells and extended to DEHP in microglial and renal models [34,35]. Structural features such as sidechain branching and hydrophobicity may modulate cellular uptake and membrane interaction [36], although, in the absence of statistically separable IC_50_ values, their contribution cannot be evaluated as a determinant of relative potency in the present data.

Several human cell lines have been widely employed *in vitro* to assess phthalate-induced metabolic activity reduction and cellular stress. HEK293 cells (human embryonic kidney cells) have served as a prototypical model for investigating the effects of phthalates on cell viability and stress response pathways [37]. In a related study, Böckers et al. examined the toxic effects of three phthalates, butyl octyl phthalate (BOP), benzyl butyl phthalate (BBP), and butyl cyclohexyl phthalate (BCP), on MCF-7 human breast cancer cells and demonstrated that these compounds bind to and activate estrogen receptor α (ERα). Such activation induced the differential expression of 15 genes implicated in tumorigenesis, cell cycle dysregulation, and adverse cancer outcomes. Collectively, these findings underscore the endocrine-disrupting potential of phthalates and their capacity to promote breast cancer progression [38].

The MTT assay revealed that the metabolic activity reduction effects of DINP, DnBP and DEHP were most distinct at the highest concentrations and declined progressively as the phthalate concentration decreased. Elevated concentrations also produced marked cellular lysis and detachment, resulting in disruption of the confluent monolayer, and this loss of cellular integrity was directly proportional to the phthalate concentration [17]. In agreement with our data, Poitou et al. reported comparable effects of DEHP, DINP, and DEHT on the viability of human endothelial cell cultures, as assessed by MTT. Their findings demonstrated substantial reductions in cell viability and a time-dependent decline in the number of viable cells, beginning as early as 48 h post-exposure [39]. Kruger et al. reported that di-butyl phthalate (DBP), butyl-benzyl phthalate (BBP), and diethylhexyl phthalate (DEHP) exposure decreased the growth of a human corneal endothelial cell line by 50 to 200 mmol/L [40].

Beyond the reduction in metabolic viability, our results indicate that phthalate exposure is accompanied by alterations in the global methylation status of NHFs. DEHP and DINP produced significant elevations in global 5-mC content (189.18% and 159.56% of control, respectively), whereas DnBP induced only a marginal, non-significant increase (120.06%). Notably, the magnitude of the methylation response did not track the extent of viability loss across the three chemicals. DnBP reduced viability comparably to DEHP and DINP yet produced no significant methylation change. This dissociation suggests that global hypermethylation is not a simple downstream consequence of exposure intensity or of cell death. We emphasize, however, that this study was not designed to establish a mechanistic relationship between these endpoints. No determinants of methylation control were measured, DNMT1, DNMT3A/3B and MeCP2 expression, DNMT enzymatic activity, and ROS/glutathione status were not assessed, and global 5-mC quantification cannot resolve locus-specific redistribution of methyl marks. These findings extend prior mechanistic work by Ghosh et al. who demonstrated that DEHP triggers global DNA hypermethylation in MCF-7 cells through selective upregulation of DNMT1 and MECP2, with downstream silencing of the cell-cycle regulator *p21* [41]. Similarly, DEHP exposure in human bronchial epithelial cells has been shown to alter *DNMT* expression in parallel with reduced viability [42], corroborating the coupling between cytotoxic and epigenotoxic effects observed in the present work. The comparatively modest effect of DnBP is noteworthy given that Sicińska et al. reported that DBP and its metabolite MBP disturbed promoter-specific methylation of tumor suppressor (*P16*, *TP53*) and proto-oncogene (*BCL2*, *CCND1*) loci in peripheral blood mononuclear cells without uniformly altering global methylation, suggesting that DnBP may act preferentially through locus-specific rather than genome-wide mechanisms [43]. These reports cannot be assumed to operate in normal human fibroblasts on the basis of the present data and are offered as context for future work rather than as an explanation of the responses observed here. The lack of a consistent correspondence between the observed metabolic activity responses and the magnitude of hypermethylation further indicates that the two endpoints varied independently across congeners. This pattern suggests that global hypermethylation is not simply related to the observed metabolic activity response under the conditions tested.

## 6 Limitations

Several limitations should be considered when interpreting these findings. First, cell viability was assessed using a single metabolic endpoint; the MTT assay measures mitochondrial dehydrogenase activity and cannot distinguish cytostatic from cytocidal effects or confirm the mode of cell death, a particular consideration for phthalates, which perturb mitochondrial function directly. Orthogonal endpoints such as LDH release, trypan blue exclusion, or annexin V/PI staining would be required to characterize the observed viability loss, and the accompanying morphological observations are qualitative. Second, global 5-mC content is a bulk measure that cannot resolve locus-specific changes. Third, no mechanistic determinants were measured, and no causal relationship between the two endpoints can be inferred. Fourth, the concentrations employed (31.25–1000 μg/mL) exceed reported human internal exposures; the study is a comparative *in vitro* screen, not a model of chronic low-dose or mixture exposure, and extrapolation to human risk is not warranted. Moreover, at the upper end of the range the highly lipophilic congeners (DEHP, log Kow ≈ 7.6; DINP, ≈ 8.8) may exceed their aqueous solubility in serum-supplemented medium, so freely dissolved concentrations may fall below nominal values, and the absolute concentrations reported should be regarded as comparative rather than exact. Finally, no matched DMSO vehicle control was included; untreated cells served as the sole negative control. As DMSO can influence both cell viability and DNA methylation even at low concentrations (≤0.20% v/v here), a residual solvent contribution to the observed effects cannot be fully excluded. The reported effects should therefore be interpreted as those of the phthalate–vehicle combination, and a matched vehicle control is a necessary refinement for future work.

## 7 Conclusion

The present study demonstrates that DEHP, DnBP, and DINP reduce the metabolic viability of normal human fibroblasts in a concentration-dependent manner, and that DEHP and DINP significantly elevate global DNA methylation whereas DnBP produces only a marginal effect. The two endpoints varied independently across chemicals, and no mechanistic link between them is claimed. These findings indicate that the effects of phthalates on the viability and epigenetic status of non-transformed human cells are phthalate-specific and identify normal human fibroblasts as a tractable model for further mechanistic investigation of phthalate-associated epigenetic change.

## CRediT authorship contribution statement

**Warqaa Y. Salih:** Data curation, Methodology, Resources, Writing – original draft. **Fikrat M. Hassan:** Conceptualization, Data curation, Supervision. **Majeed A. Sabbah:** Resources, Supervision, Validation.

## Ethics declaration

Written informed consent to take part in the study and to publish the article has been obtained from all participants or their legal representatives. The privacy rights of participants have been observed.

This study included organ or tissue donors. This study includes human biological material and consent was obtained by donors, or their next of kin or legal representatives, for use in this study and for publication of the article. The samples used in this research were not sourced from executed prisoners or prisoners of conscience.

This study was performed in compliance with relevant laws, regulatory frameworks and guidelines where the research took place. This study was conducted in accordance with Helsinki (2013 revision). This study was approved by the Institutional Ethics Committee of the ICCRMG. (Approval No. approval no. ICCRMG-79, dated 1 November 2024)

## Funding

This work was prepared without any financial support.

## Declaration of competing interests

The authors declare that there are no conflicts of interest.

## Data Availability

No data was used for the research described in the article.
